# Exploring Synergistic Combinations in Extended and Pan-Drug Resistant (XDR and PDR) Whole Genome Sequenced *Acinetobacter baumannii*

**DOI:** 10.3390/microorganisms11061409

**Published:** 2023-05-26

**Authors:** Munawr AL Quraini, Zaaema AL Jabri, Hiba Sami, Jaspreet Mahindroo, Neelam Taneja, Zakariya AL Muharrmi, Ibrahim AL Busaidi, Meher Rizvi

**Affiliations:** 1 Department of Microbiology and Immunology, College of Medicine and Health Sciences, Sultan Qaboos University, Muscat 123, Oman; munquraini@outlook.com (M.A.Q.); zaeema@squ.edu.om (Z.A.J.); almuharrmi@gmail.com (Z.A.M.); 2 Department of Microbiology, Jawahar Lal Nehru Medical College, AMU, Aligarh 202001, India; hibasamizafar@gmail.com; 3 Department of Medical Microbiology, Post Graduate Institute of Medical Education and Research, Chandigarh 160012, India; jassi.mahindroo12@yahoo.com (J.M.); drneelampgi@yahoo.com (N.T.); 4 Infectious Diseases Unit, Sultan Qaboos University Hospital, Muscat 123, Oman; ibrahimbusaidi@gmail.com

**Keywords:** in vitro synergy, *Acinetobacter baumannii*, checkerboard assay, meropenem, time kill assay, fosfomycin

## Abstract

**Background:** The diminishing antimicrobial options for the treatment of XDR and PDR *Acinetobacter baumannii* is an increasing concern. In this study, we assessed the in vitro synergy of the fosfomycin (FOS) with meropenem (MEM), amikacin (AK), tigecycline (TGC), and colistin (CL) in whole genome sequenced isolates. **Methods:** Non-replicate whole genome sequenced (illumina next-generation sequencing platform, Clevergene, India), *A. baumanii* (7 XDR, 1PDR) were subjected to in vitro synergy testing by checkerboard (CB) and time kill assay (TKA) after MIC determination, with glucose-6-phosphate being incorporated in all runs. FOS was used as a cornerstone drug in four combinations and colistin in one. ResFinder, MLST, PlasmidFinder, and CSIPhylogeny tools were used. **Results:** Mortality occurred in three patients. Diverse MLST were observed, ST-1962 (3 isolates) and one each of ST2062, ST2063, ST1816, ST1806, ST234. FOS MICs ranged from 32 to 128 mg/L, MEM MIC: 16–64 mg/L, TGC MIC: ≤2–≤4 mg/L and AK MIC: >512 mg/L. CL: MIC range, 0.25–≤2 mg/L, PDR MIC > 16 mg/L. Synergy results by CB: FOS-MEM: synergy in ⅞ (90%) isolates. Synergy lowered MEM MICs to susceptibility breakpoints in 6/8 cases. CL-MEM: Excellent synergy (3/3) isolates. FOS-AK: Indifference in ⅞, antagonism ⅛ (AK-susceptible isolate). FOS-TGC: Partial synergy (PS) in 8/8 (TGC MIC dropped to ≤0.25 mg/L in 3/8). In the PDR isolate, synergy was seen in FOS-MEM, CL-MEM, PS in FOS-CL, FOS-TGC, indifference in FOS-AK. TKA: Excellent synergy was observed with FOS-MEM from 4 h, while FOS-AK and FOS-TGC demonstrated synergy at 24 h. Synergy was achieved despite presence of widespread resistance markers against aminoglycosides (*AacAad*, *AadA*, *AadB*, *Aph3″Ia*, *ArmA*, *Arr*, *StrA*, *StrB*), beta-lactams (ADC, BlaA1, BlaA2, Zn-dependent_hydrolase, OXA-23, OXA-51, PER-1,TEM-1D, CARB-5, Mbl), sulphonamides (SulII, SulI), phenicols (*CatBx*, *CmlA*), macrolides (*MphE*, *MsrE*) and tetracycline (*TetB*) were widespread. Carbapenemase, CARB-5 was present in one isolate. Beta-lactamase genes OXA-23, OXA-51, *Bla*A2, Zn-dependent_hydrolase, ADC, Mbl and macrolide resistance genes *MphE*, *MsrE* were present in all 8 isolates. **Conclusions:** FOS-MEM and CL-MEM are promising combinations against *A. baumannii*. Synergy of FOS-MEM in intrinsically resistant *A. baumannii* shows that this antibiotic combination might be useful in treating such XDR and PDR pathogens.

## 1. Introduction

*Acinetobacter baumannii*, an aerobic, Gram-negative, opportunistic coccobacilli, is frequently linked to nosocomial infections and is a predominant pathogen in the intensive care unit (ICU). It has the potential to cause difficult to treat bacteremia, ventilator-associated pneumonia, meningitis, and urinary tract infections due to the acquisition of multiple drug resistance [1]. Multiple pathways are responsible for the acquisition of antimicrobial resistance [2]. Conventional antimicrobials are frequently rendered ineffective by multiple inherent and acquired resistance mechanisms, which leads to the rise of extensively drug-resistant (XDR) strains [3]. An isolate is classified as XDR when it is non-susceptible to at least one agent in all but two or fewer antimicrobial categories [3]. Pan drug-resistance (PDR) implies resistance to all agents in all antimicrobial classes [3]. Due to the paucity of new antimicrobials, finding effective antibiotic combinations to treat XDR and PDR *A. baumanii* infections is crucial. Extended and pan drug-resistant (XDR and PDR) strains of *A. baumannii* are of particular concern as they are resistant to nearly all available antibiotics, leaving clinicians with limited treatment options. Exploring synergistic combinations of antibiotics is an important area of research for combating XDR and PDR *A. baumannii* strains. Synergistic combinations of antibiotics involve using two or more drugs that work together to enhance their effectiveness, allowing for better control of bacterial growth and reducing the likelihood of antibiotic resistance. This approach has the potential to improve treatment outcomes for patients with XDR and PDR *A. baumannii* infections and can help slow the spread of antibiotic-resistant strains [4]. Tests of in vitro antimicrobial synergy can provide important insight into which combinations will be successful in treating these challenging infections. Fosfomycin’s exceptional features, such as its ability to reach high plasma concentrations and penetrate tissues effectively, low cross-resistance [5], and absence of nephrotoxicity [6], make it a desirable primary medication to use in conjunction with other antibiotics. Moreover, in recent years, whole genome sequencing (WGS) has emerged as a powerful tool for identifying genetic mechanisms underlying antibiotic resistance and identifying potential new treatment strategies This study aimed to evaluate the in vitro synergistic interactions of fosfomycin with meropenem, colistin, tigecycline, and amikacin against XDR and PDR *Acinetobacter baumannii* using checkerboard and time-kill assays. We further assessed whether synergy would lead to lowering of the MICs to clinical breakpoints or below.

## 2. Material and Methods

A ten-month study, spanning from September 2019 to June 2020, this study was carried out at the Sultan Qaboos University Hospital’s Department of Microbiology and Immunology in collaboration with the Sultan Qaboos University Hospital’s Department of Medicine in Muscat, Sultanate of Oman, the Department of Medical Microbiology at the Postgraduate Institute of Medical Education and Research in Chandigarh, India. Prior to the start of the study, approval was obtained from the Medical Research Ethics Committee of the College of Medicine and Health Sciences at Sultan Qaboos University, Muscat, Oman.

Eight non-duplicate genotyped (7 XDR and 1 PDR) strains of *Acinetobacter baumannii* recovered from urinary tract, wound, respiratory tract, and bloodstream infections were assessed for synergy and molecular determinants of resistance. In vitro synergy testing by checkerboard (CB) and time kill assay (TKA) was performed after MIC determination, with glucose-6-phosphate being incorporated in all runs. FOS was used as a cornerstone drug in four combinations and colistin in one. Cepheid XpertCarba-R assay was used for preliminary genotypic characterization (Cepheid, Sunnyvale, CA, USA). The strains were cryopreserved and kept at −40 °C in sterile CryoBeads (Mast Diagnostics, UK) that included glycerol and a hypertonic additive along with a cryopreservative fluid.

### 2.1. Whole Genome Sequencing (WGS)

The genomic DNA of all 8 *A. baumanii* isolates was purified using a Qiagen kit (QIAquick PCR and Gel Cleaning Kit, 2018) in accordance with the manufacturer’s instructions after being isolated from 18 to 24 h old cultures using the traditional phenol-chloroform procedure [4]. For whole genome sequencing, extracted DNA was transferred to Clevergene Biocorp Pvt Ltd., Bangalore, India (Illumina next-generation sequencing). The Centre for Genomic Epidemiology, 2020 website’s tools were used to retrieve the sequences and analyze them. The acquired antimicrobial resistance genes, multilocus sequence typing (MLST), plasmids, and bacterial relatedness were all investigated using the ResFinder, MLST, PlasmidFinder, and CSIPhylogenyprogrammes, respectively. By locating and filtering high-quality SNPs, the CSIPhylogeny programme was able to detect differences between the generated sequence data (FASTA files) (z-score higher than 1.96 for all SNPs). The CARD bioinformatics, 2020 website was used to detect resistance genes.

### 2.2. MIC Determination

The following powders were purchased from Sigma-Aldrich Chemical Co., Saint Louis, MO, USA: fosfomycin (FOS), colistin sulphate (CL), amikacin (AK); meropenem (MEM), from the United States Pharmacopoeia, and tigecycline (TGC), from the European Pharmacopoeia, and stored at 4 °C or <−20 °C until use as per the manufacturers’ recommendations. The following strains ATCC 25922 (*Escherichia coli*), ATCC 27853 (*Pseudomonas aeruginosa*), and ATCC 29212 (*Enterococcus faecalis*) were used for quality control. The MICs were determined by two methods: the agar dilution method (AD) for fosfomycin on MHA plates (150-mm diameter) supplemented with 25 mg glucose-6-phosphate (G-6-P/L) and the broth microdilution (BMD) method (Becton Dickinson) against FOS, AK, MEM, TGC, and CL as described by CLSI guidelines (M07-A10, Vol.35 No.2, 2015) [7].

### 2.3. Antimicrobial Synergy Testing

Synergy testing was performed with FOS as the cornerstone drug: FOS-MEM, FOS-CL, FOS-TGC, and FOS-AK. We used the checkerboard assay for all combinations and the time-kill assay for representative strains for verifying our results as per Rizvi et al., 2013 [8].

### 2.4. Checkerboard Assay

The broth microdilution checkerboard (BMC) assay was conducted in duplicate. The MIC for each isolate determined the range of antibiotic concentrations to be utilised in the checkerboard assay. The concentration of the antibiotics ranged from ≤1/32 × MIC to 1 × MIC. FICI = FIC A in combination/MIC A alone, FIC B = MIC B in combination/MIC B alone, and FICI = (MIC of drug A in combination/MIC of drug A alone) + (MIC of drug B in combination/MIC of drug B alone) were used to calculate the type of interactions between the antimicrobials. FICI of 0.5 implied synergy, FICI > 0.5 to 1, partial synergism, and FICI > 1 to 4 indicated indifference, and FICI > 4 antagonism [7].

### 2.5. Time-Kill Assay

The time-kill assay is a dynamic assessment of the effect of the antimicrobial combination on the bacterial strain at several time periods (2, 4, 6, 24 h), while there is just a one-time assessment in checkerboard. It sheds light on whether the interaction is bactericidal, bacteriostatic, or if regrowth occurred at 24 h. To confirm the synergistic or additive reactions observed using the checkerboard approach as previously described, TKA was conducted in representative strains [7]. The time-kill curves were created by plotting the bacterial cell counts for a. growth control, b. each individual antibiotic, and c. the antimicrobial combination against time. A 2-log_10_ CFU/mL decrease in bacterial growth signified synergism, antagonism was defined as a 2-log_10_ CFU/mL increase in bacterial growth in the combination compared to the highest active single drug, while indifference was defined as a <2-log_10_ increase or decrease in colony count. A 3-log_10_ CFU/mL drop in bacterial counts was considered a bactericidal outcome [9].

### 2.6. Statistical Analysis

The IBM Statistical Package for the Social Sciences (SPSS) was used to analyse the data. Paired sample *t*-test was used to determine whether the MIC reduction in the combination interaction (alone vs combined) was significant. A *p*-value of ≤0.05 was considered statistically significant.

## 3. Results

The XDR and PDR *A. baumannii* were isolated from the respiratory tract (n = 5), wound (n = 3), and bloodstream (n = 1) infections. Male patients predominated (n = 6/8), of which three expired.

### 3.1. Minimal Inhibitory Concentrations (MIC) of Fosfomycin, Meropenem, Tigecycline, Colistin and Amikacin in Acinetobacter baumannii

Fosfomycin MIC ranged from 32 to ≥128 mg/L (Table 1), MEM MIC: 16–64 mg/L, TGC MIC: ≤2 to ≤4 mg/L and AK MIC: >512 mg/L. CL: MIC range, 0.25 to ≤2 mg/L, PDR MIC > 16 mg/L. As per EUCAST, the MICs of fosfomycin for all but two isolates were in the resistant range (>32 mg/L) by agar dilution. All isolates were resistant to MEM (16–64 mg/L). All isolates except one were resistant to TGC (2 to ≥4 mg/L) and AK (MIC >512 mg/L). All isolates were susceptible to colistin (MIC range, 0.25 to ≤2 mg/L) except for the PDR isolate (16 mg/L).

### 3.2. Synergy Outcomes

Synergy (SY), partial synergy (PS), indifference (IN), and antagonism (AN) against fosfomycin in different antimicrobial combinations is shown in Table 2. FOS-MEM combination showed synergy in ⅞ (90%) isolates. Synergy lowered MEM MICs to susceptibility breakpoints in 6/8 cases. CL-MEM combination showed excellent synergy (3/3) isolates. FOS-AK combination showed indifference in ⅞ and antagonism in an AK-susceptible isolate. FOS-TGC combination showed partial synergy in all isolates (TGC MIC dropped to ≤0.25 mg/L in 3/8 isolates).

### 3.3. Outcome of Fosfomycin-Meropenem Combination by Checkerboard Assay

Out of 8 isolates, 7 (88%) showed effective synergistic interactions at 0.25 MIC FOS + 0.25 MIC MEM (Table 2). Although the synergistic interactions could not bring down the MEM MICs to the susceptible range, i.e., ≤2 mg/L, it did drop to 8–16 mg/L.

### 3.4. Assessment of Fosfomycin-Meropenem Interactions of A. baumannii by Time-Kill Assay

**Figure 1:**
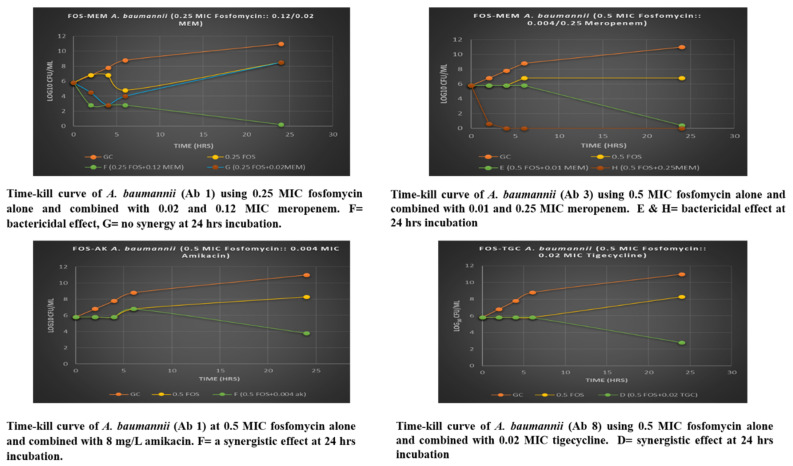
Time Kill Analysis of *A. baumannii* with fosfomycin alone and in combination with meropenem, amikacin and tigecycline.

On assessing FOS-MEM by TKA in the representative strain, excellent synergy was observed with FOS-MEM from 4 h onwards, while synergy was observed from 24 h onwards with FOS-AK and FOS-TGC (Figure 1). Synergy was observed despite the widespread presence of resistance markers. Time-kill assay was conducted to confirm the observed synergy at 0.25 MIC FOS and partial synergy at 0.5 MIC FOS with extremely low meropenem (0.5 mg/L). Bacterial count in the growth control increased gradually by one log_10_ every 2 h through 6-h incubation and more than five log_10_ at 24 h. At 1 MIC, fosfomycin alone and in combination with 0.5 MIC meropenem (64 mg/L) reduced the bacterial growth to zero counts at all time intervals.

The antimicrobial effect of the combination was proportionally related to a higher meropenem concentration. The bactericidal activity increased greatly at 0.25 MIC fosfomycin with a higher dose of meropenem (0.12 MIC MEM = 16 mg/L) and demonstrated bactericidal effect at 2–24 h incubation (Table 3). The ratio of 0.25 MIC fosfomycin with low meropenem concentration (2 mg/L) had a synergistic effect at 2 h and bactericidal at 4 h; however, the growth increased significantly to 2–3 log_10_ at 24 h. The combination was more potent and its effect was further augmented in the ratio of 0.5 MIC FOS and a high MEM dose (0.25 MEM MIC= 32 mg/L), which brought down the bacterial population to constant zero counts at 4–24 h incubation. At an extremely low meropenem concentration (0.01 MIC MEM= 0.5 mg/L), it was interesting to observe that there was bactericidal activity at 24 h incubation.

### 3.5. Synergy in PDR Strains

PDR strain (Ab1) demonstrated excellent synergy in FOS-MEM, CL-MEM, PS in FOS-CL, FOS-TGC and indifference in FOS-AK (Table 2).

### 3.6. Identification of STs

The isolates surprisingly exhibited varying MLSTs as seen in Table 4, except for two isolates, which belonged to ST-1962; the other five had a different MLST (ST2062, ST2063, ST1816, ST1806, ST234).

### 3.7. Antibiotic Resistance Genes

We performed whole genome sequencing on XDR and PDR *A. baumannii* isolates and identified several antibiotic resistance genes. Resistance to aminoglycosides (*AacAad*, *AadA*, *AadB*, *Aph3″Ia*, *ArmA*, *Arr*, *StrA*, *StrB*), beta-lactams (ADC, BlaA1, BlaA2, Zn-dependent_hydrolase, OXA-23, OXA-51, PER-1, TEM-1D, CARB-5, Mbl), sulphonamides (*SulII*, *SulI*), phenicols (*CatBx*, *CmlA*), macrolides (*MphE*, *MsrE*), and tetracycline (*TetB*) was widespread. Carbapenemase, CARB-5 was present in one isolate. Beta-lactamase genes OXA-23, OXA-51, *Bla*A2, Zn-dependent_hydrolase, ADC, *Mbl* and macrolide resistance genes *MphE*, *MsrE* were present in all isolates.

## 4. Discussion

The diminishing antimicrobial options for the treatment of XDR and PDR *A. baumanii* are a huge challenge. As we inexorably inch towards the post-antibiotic age, when treating critically ill patients, one has to resort to drugs of last resort such as fosfomycin, colistin, tigecycline, and carbapenems. Ceftazidime-avibactam was introduced in our facility subsequent to our study and was therefore not tested. It is also a harsh reality that a large population group does not have access to the newly introduced beta-lactam/beta-lactamase inhibitors, cefiderocol, eravacycline, and plazomycin. In such populations, the drug combinations that we have tested are more accessible. Empirical monotherapy with these antimicrobials is usually not advisable in XDR *A. baumanii*, as there are chances of the development of resistance and a possibility of treatment failure [10]. Management of PDR, on the other hand, undoubtedly entails a cocktail of antimicrobials [11]. Several studies have pointed to the utility of using combinations of antimicrobial agents to arrive at a synergistic effect, thus substantially reducing the risk of inappropriate empirical therapy on the one hand and the development of resistance on the other [12,13].

All the XDR isolates in this study were susceptible to only one (colistin) or a maximum of two antibiotic classes, except the MDR *A. baumannii* (Ab 8), which was sensitive to colistin, amikacin, and tigecycline. In this study, excellent synergy was observed with FOS-MEM and CL + MEM combinations against *A. baumannii* XDR and PDR isolates despite the widespread presence of resistance markers. Fosfomycin alone has excellent bactericidal activity (zero colonies) against XDR and PDR *A. baumanii* isolates, which began as early as 2 h and the activity continued as late as 24 h. A study from Australia [14] also reported that the in vitro activity of FOS against the carbapenem-resistant *A. baumannii*(CR-AB) isolates was enhanced by the addition of MEM. Overall, our results suggest that the fosfomycin-meropenem combination may have potential as a treatment option for XDR and PDR *A. baumannii* infections, and further studies are needed to validate this finding in clinical settings. Similarly, Singkham-in and Chatsuwan, 2018 reported the synergism of fosfomycin-imipenem in 65.2% of OXA-23-producing CR *A. baumannii* [15].

Beta-lactamase genes OXA-23, OXA-51, BlaA2, Zn-dependent_hydrolase, ADC, Mbl, and macrolide resistance genes MphE, MsrE were present in all 8/8 isolates. Other resistance genes were against aminoglycosides (*Aac*, *Aad*, *AadA*, *AadB*, *Aph3″Ia*, *ArmA*, *Arr*, *StrA*, *StrB*), beta-lactams (ADC, BlaA1, BlaA2, Zn-dependent_hydrolase, OXA-23, OXA-51, PER-1, TEM-1D, CARB-5, Mbl), sulphonamides (*SulII*, *SulI*), phenicols (*CatBx*, *CmlA*), macrolides (*MphE*, *MsrE*), and tetracycline (*TetB*). Interestingly, one isolate, Ab 8, was found to harbour the carbapenemase gene CARB-5, which is a cause for concern as carbapenems are often used as a last-resort treatment for *A. baumannii* infections. Overall, our study highlights the extent of antibiotic resistance in XDR and PDR *A. baumannii* isolates and emphasises the urgent need for novel treatment strategies to combat this public health threat.

FOS-AK combination displayed a disappointing lack of synergy against *A. baumannii* isolates. Indifferent interactions were seen against all isolates by checkerboard assay. The low activity of this combination could be attributed to high-level aminoglycoside resistance (HLAR) in *A. baumannii*, [16] the prevalence of aminoglycoside-modifying enzymes, and the over-expression of efflux pumps such as adeABC [17,18]. A Study by Leite et al., 2016 [19] demonstrated the synergistic effects of this combination in both colistin-susceptible and resistant OXA-23-like *A. baumannii* isolates using the time-kill assay. Similar to our findings, indifferent outcomes were observed by the checkerboard. The colistin-resistant isolates in their study displayed the same CL MIC range (8–64 mg/L) and FOS MIC90 of 128 mg/L. However, they did not report the individual strain MIC after combination, which would have been useful to understand when to expect synergy in this combination. In our study, the observed antagonistic interactions in all amikacin susceptible *A. baumannii* isolates suggests that the monotherapy by either antibiotic would be more helpful for patient management, though other studies have reported the synergistic effect of fosfomycin-amikacin combination [20,21].

The MIC_50_ of tigecycline in the XDR *A. baumannii* was 4 mg/L, while MIC_90_ of fosfomycin was higher at 128 mg/L. Despite the high MIC_90_, all isolates (100%) of *A. baumannii* displayed partial synergistic interactions at ≤0.03 MIC FOS + 0.5 MIC TGC (1–2 mg/L). Compared to fosfomycin-amikacin, a high rate of antagonism was observed in the fosfomycin-tigecycline combination against *A. baumannii* isolates. The time-kill assay confirmed the synergistic interaction at this ratio in the representative isolate and showed synergy (>2 log_10_ decline) at 24 h incubation. Thus, 0.5 MIC fosfomycin combined with very low tigecycline concentration (0.06 mg/L) had a substantial synergistic effect over 0.5 MIC fosfomycin alone at 24 h incubation but no bactericidal activity was observed.

The colistin-meropenem combination displayed excellent synergism in all *A. baumannii* (3/3), with the best outcomes in the PDR (Ab 1). Effective synergistic interactions with a relatively low meropenem MIC (≤32 mg/L) of OXA-51/23 XDR-*A. baumannii* strains were demonstrated in vivo using a Murine Thigh-Infection Model by Fan et al. 2016 [22]. This could explain the notable outcome in the PDR Ab isolate, which had a meropenem MIC of 16 mg/L compared to other isolates (MIC 32 mg/L), although it must be noted that the colistin MIC in this case was higher (8 mg/L). A study from China also demonstrated the superiority of colistin-meropenem against CR *A. baumannii* with an MIC of ≥32 mg/L by checkerboard and static time-kill assays [23]. In their study, an effective dosage regimen of 2 g meropenem daily via 3-h infusion combined with steady-state 1 mg/L colistin suppressed bacterial growth at 24 h with a 2-log_10_ decrease. This finding is quite similar to our partial synergistic outcomes in the checkerboard at 0.5–1 mg/L MIC MEM + 0.25–1 mg/L MIC CL. The possible mechanism behind this synergism is that colistin interferes with the outer membrane, causing disruption to its permeability and subsequently allowing higher concentrations of meropenem (inhibition of peptidoglycan) to enter the bacterial cells so that the resistant bacteria become more susceptible [24]. Maifiah et al. 2017 discovered that colistin-doripenem combination kills *A. baumannii* synergistically in a time-dependent manner by perturbing different key metabolic pathways necessary for bacterial survival using liquid-chromatography mass spectrometry (LC-MS) [25].

FOS-CL combination demonstrated disappointingly indifferent interactions in the PDR isolates. On the other hand, excellent synergy was observed in PDRs with FOS-MEM (FICI ≤ 0.40) and FOS-AK (FICI ≤ 0.50), while antagonism was noted with FOS-TGC combination. Further studies are needed to confirm the efficacy of these antibiotic combinations in vivo and to determine the optimal dosages and treatment regimens.

The variation in MLSTs among the isolates in this study is an interesting finding and could suggest the presence of diverse sources of infection or the acquisition of resistance genes through horizontal gene transfer. MLST is a widely used method for molecular typing of *A. baumannii* and helps in identifying the clonal relatedness among the isolates [26]. The fact that two isolates belonged to the same ST, ST-1962, indicates a possible clonal outbreak. However, the presence of different STs among the other isolates suggests the possibility of multiple sources of infection or acquisition of resistance genes through horizontal gene transfer. Further studies are required to determine the epidemiology and genetic relatedness among the isolates.

## 5. Conclusions

Our study demonstrated that the FOS and MEM combination could be a useful option to treat XDR and PDR *A. baumannii*. Synergy of FOS-MEM in intrinsically resistant *A. baumannii* shows that this antibiotic combination might be useful in treating CR-AB isolates. To further support the findings, a larger preclinical or clinical investigation is essential.

## Figures and Tables

**Table 1:** Minimum inhibitory concentrations (MICs) of fosfomycin, meropenem, tigecycline, colistin, and amikacin against *A. baumannii* isolates (n = 8).

Isolate	MIC Value (mg/L)					
	Fosfomycin		Meropenem	Tigecycline	Amikacin	Colistin
	AD *	BMD ^#^	BMD ^#^	BMD ^#^	BMD ^#^	BMD ^#^
**Ab1 (PDR)**	128	128	16	2	>512	16
**Ab 2**	128	64	32	2	>512	0.5
**Ab 3**	128	≤128	32	4	>512	1
**Ab 4**	32	64	32	4	>512	1
**Ab 5**	32	64	32	2	>512	1
**Ab 6**	≤128	128	32	4	>512	1
**Ab 7**	64	32	32	4	>512	0.25
**Ab 8**	64	64	64	≤0.5	≤8	≤1

* AD: agar dilution, ^#^ BMD: broth microdilution, Ab: *A. baumannii*.

**Table 2:** Outcomes of various antibiotic combinations using the Checkerboard assay in XDR and PDR *A. baumannii* isolates (n = 8).

Isolate	Fos MIC (mg/L)		Fold Decline	MEM MIC (mg/L)		Fold Decline	FICI (x̄)
	Alone	Combined with MEM		Alone	Combined with FOS		
**Ab1**	128	32	4	16	4	4	0.50 (S)
**Ab2**	64	1	≥64	32	16	2	0.52 (PS)
**Ab3**	64	16	4	32	8	4	0.50 (S)
**Ab4**	64	16	4	32	8	4	0.50 (S)
**Ab5**	64	16	4	32	8	4	0.50 (S)
**Ab6**	128	32	4	32	8	4	0.50 (S)
**Ab7**	32	8	4	32	8	4	0.50 (S)
**Ab8**	64	16	4	64	16	4	0.50 (S)
	**FOS MIC (mg/L)**			**AK MIC (mg/L)**			
	**alone**	**Combined with AK**		**alone**	**Combined with FOS**		
**Ab1**	128	128	0	>1024	≤8	≥256	1.00 (IN)
**Ab2**	128	128	0	>1024	≤8	≥256	1.00 (IN)
**Ab3**	128	128	0	>1024	≤8	≥256	1.00 (IN)
**Ab4**	64	64	0	1024	≤4	≥256	1.00 (IN)
**Ab5**	64	64	0	1024	≤4	≥256	1.00 (IN)
**Ab6**	128	128	0	1024	≤4	≥256	1.00 (IN)
**Ab7**	32	32	0	>1024	≤4	≥512	1.00 (IN)
	**MEM MIC (mg/L)**			**CL MIC (mg/L)**			
	**alone**	**Combined with COL**		**alone**	**Combined with MEM**		
**Ab 1**	16	0.5	32	8	≤1	≥8	0.16 (S)
**Ab 2**	32	4	8	≤1	0.25	4	0.37 (S)
**Ab 3**	32	8	4	1	0.25	4	0.50 (S)
	**FOS MIC (mg/L)**			**TGC MIC (mg/L)**			
	**alone**	**Combined with TGC**		**alone**	**Combined with FOS**		
**Ab 1**	128	≤2	≥64	2	1	2	0.52
**Ab 2**	128	≤4	≥32	2	1	2	0.53
**Ab 3**	128	≤4	≥32	4	2	2	0.53
**Ab 4**	64 64	≤1 32	≥64 2	4 4	2 0.25	2 16	0.52 0.56
**Ab 5**	64 64	≤1 32	≥64 2	2 2	1 ≤0.06	2 ≥32	0.52 0.53
**Ab 6**	128 128	≤1 64	≥128 2	4 4	2 ≤0.06	2 ≥64	0.51 0.52
**Ab 7**	32	≤1	≥32	4	2	2	0.53

Abbreviation: Ab = *A. baumannii*, FICI = fractional inhibition concentration index, x̄ = mean value, S = synergy, PS = partial synergy, MIC = minimal inhibition concentration, x̄ = mean value. FOS, fosfomycin; IMI, imipenem; MEM, meropenem; CL, colistin; AK, amikacin; TGC, tigecycline; synergy (SY), partial synergy (PS), indifference (IN) and antagonism (AN).

**Table 3:** Summary of time-kill and checkerboard assessments of different combinations in different bacteria (n = 8).

Combination	[(FOS) + (MEM)] mg/L	6-h Effect	24-h Effect	24-h log_10_ Killing ∆ 24 h	Checkerboard
Fosfomycin + Meropenem	Isolate Ab 3				
0.25 FOS + 0.02 MEM	(32, 2)	Synergy	None	>+2	Growth
0.25 FOS + 0.12 MEM	(32, 8)	Bactericidal	Bactericidal	**>−5**	Synergy
0.5 FOS + 0.01 MEM	(64, 0.5)	**None**	Bactericidal	**>−5**	Growth
0.5 FOS + 0.25 MEM	(64, 16)	Bactericidal	Bactericidal	**−6**	Partial synergy

**Fosfomycin + Amikacin**	**Isolate Ab 1**				
0.5 FOS + 0.004 AK	(64, 8)	**None**	Synergy	**−2**	Growth

**Fosfomycin + Tigecycline**	**Isolate Ab 4**				
0.5 FOS + 0.02 TGC	(64, 0.06)	**None**	Bactericidal	**−3**	Partial synergy

Abbreviation: FOS = fosfomycin, MEM = meropenem, TGC = tigecycline, AK = amikacin, Ab = *A. baumannii*, Bactericidal effect = ≥3 log_10_ reduction in CFU/mL after 24 h compared with the starting inoculum (0 h). Synergy = ≥2 log_10_ reduction in CFU/mL after 24 h compared with in the antibiotic alone. Difference (**∆**) in bacterial concentration in log_10_ CFU/mL at 24 h compared with the starting inoculum. Bactericidal effect (≥3 log_10_ reduction in CFU/mL after 24 h) and synergy (≥2 log_10_ reduction in CFU/mL at 24 h with the combination as compared with the most active single drug). Checkerboard FICI values of x ≤ 0.5 (synergism) and 0.5 < x < 1 (partial synergy).

**Table 4:** Multi-Locus Sequence Typing (MLST) of *A. baumannii* isolates (n = 8).

Sample	ST	gltA	gyrB	gdhB	recA	cpn60	gpi	rpoD	Mismatches	Uncertainty	Depth	maxMAF
**Ab1**	2062	1	17	189	2	2	108	3	0	-	197.8256	0.016667
**Ab2**	2063	1	17	3	2	2	108	3	0	-	187.7057	0.013793
**Ab3**	1816	1	3	189	2	2	96	3	0	-	161.3086	0.015625
**Ab4**	1962	1	3	189	2	2	140	3	0	-	234.2391	0.00885
**Ab5**	1962	1	3	189	2	2	140	3	0	-	210.5916	0.011364
**Ab6**	1806	1	3	189	2	2	97	3	0	-	179.503	0.012422
**Ab7**	234	21	48	58	42	36	109	4	0	-	258.423	0.013263

## Data Availability

Not applicable.
